# CD4^+^ T Cells Expressing Latency-Associated Peptide and Foxp3 Are an Activated Subgroup of Regulatory T Cells Enriched in Patients with Colorectal Cancer

**DOI:** 10.1371/journal.pone.0108554

**Published:** 2014-09-30

**Authors:** Jayashri Mahalingam, Chun-Yen Lin, Jy-Ming Chiang, Po-Jung Su, Yu-Yi Chu, Hsin-Yi Lai, Jian-He Fang, Ching-Tai Huang, Yung-Chang Lin

**Affiliations:** 1 College of Medicine, Chang Gung University, Kweishan, Taoyuan, Taiwan; 2 Department of Gastroenterology-Hepatology, Linkou Medical Centre, Chang Gung Memorial Hospital, Kweishan, Taoyuan, Taiwan; 3 Colorectal Surgery Section, Department of Surgery, Linkou Medical Centre, Chang Gung Memorial Hospital, Kweishan, Taoyuan, Taiwan; 4 Department of Hematology-Oncology, Linkou Medical Centre, Chang Gung Memorial Hospital, Kweishan, Taoyuan, Taiwan; 5 Department of Infectious Diseases, Linkou Medical Centre, Chang Gung Memorial Hospital, Kweishan, Taoyuan, Taiwan; New York University, United States of America

## Abstract

Latency-associated peptide (LAP) - expressing regulatory T cells (Tregs) are important for immunological self-tolerance and immune homeostasis. In order to investigate the role of LAP in human CD4^+^Foxp3^+^ Tregs, we designed a cross-sectional study that involved 42 colorectal cancer (CRC) patients. The phenotypes, cytokine-release patterns, and suppressive ability of Tregs isolated from peripheral blood and tumor tissues were analyzed. We found that the population of LAP-positive CD4^+^Foxp3^+^ Tregs significantly increased in peripheral blood and cancer tissues of CRC patients as compared to that in the peripheral blood and tissues of healthy subjects. Both LAP^+^ and LAP^−^ Tregs had a similar effector/memory phenotype. However, LAP^+^ Tregs expressed more effector molecules, including tumor necrosis factor receptor II, granzyme B, perforin, Ki67, and CCR5, than their LAP^−^ negative counterparts. The in vitro immunosuppressive activity of LAP^+^ Tregs, exerted via a transforming growth factor-β–mediated mechanism, was more potent than that of LAP^−^ Tregs. Furthermore, the enrichment of LAP^+^ Treg population in peripheral blood mononuclear cells (PBMCs) of CRC patients correlated with cancer metastases. In conclusion, we found that LAP^+^ Foxp3^+^ CD4^+^ Treg cells represented an activated subgroup of Tregs having more potent regulatory activity in CRC patients. The increased frequency of LAP^+^ Tregs in PBMCs of CRC patients suggests their potential role in controlling immune response to cancer and presents LAP as a marker of tumor-specific Tregs in CRC patients.

## Introduction

Immunosuppressive functions of a specialized subset of T cells are vital for immune regulation. Regulatory T cells (Tregs) play a central role in the maintenance of peripheral self-tolerance and immune homeostasis [1]–[3]. Several lines of evidence suggest that forkhead transcription factor (Foxp3)-expressing CD4^+^ Tregs are heterogeneous in their development and functions. Thus, natural Tregs refer to CD4^+^Foxp3^+^ Tregs of thymic origin, whereas induced Tregs (iTregs) are a T cell population peripherally converted from CD4^+^Foxp3^−^ T cells [4]–[6]. Huehn et al. were the first to demonstrate the existence of distinct subsets of Tregs, naïve Tregs and effector memory Tregs, based on the expression of CD103, a receptor of α_E_ integrin that guides T cells to inflamed sites [7]. The immunomodulatory function of activated or effector Tregs related to the expression of a variety of molecules such as chemokine receptors CCR6 and CCR5, cytotoxic T lymphocyte antigen-4 (CTLA-4), and tumor necrosis factor receptor (TNFR) II was then investigated in chronic inflammatory diseases, graft-versus-host disease, and tumors [8]–[15]. Sakaguchi et al. further delineated the role of Foxp3^+^ CD4^+^ Tregs based on the expression of CD45RA and Foxp3 and divided CD4^+^ Foxp3^+^ Tregs into three phenotypically and functionally distinct subgroups, namely non-suppressive, resting, and activated Tregs; the latter are believed to act as suppressors of immune response and mediators of immune hemostasis [16]. We had previously adopted this classification and found that in colon cancer patients, only the activated and not the naïve Tregs accumulated in the tumor site, suppressed effector T cell proliferation in vitro, and correlated with tumor progression [17]. We have suggested that, given the differential regulatory activity of human Tregs, it is necessary to separate Foxp3^+^ Tregs into functional subgroups and to target a specific Treg subpopulation in order to ensure successful immunotherapy.

A number of studies have demonstrated that transforming growth factor (TGF)-β plays a critical role in the immunosuppression exerted by Foxp3^+^ Tregs [18]–[22]. TGF-β in combination with IL-2 potently induces the differentiation of naïve Tregs into functional Foxp3^+^ iTregs [19]. Latency-associated peptide (LAP) is the N-terminal pro-peptide of the TGF-β precursor that non-covalently binds to TGF-β, forming a latent TGF-β complex and facilitating the release of TGF-β1 into the extracellular matrix [23]; subsequently, the activated TGF-β promotes the conversion of naïve Tregs to iTregs and mediates Treg-associated immunosuppression [24]. LAP is expressed on the cell membrane of many immune cells, including Tregs, and participates in immune regulation. TGF-β–dependent LAP-expressing Tregs have demonstrated suppressive ability in mice and humans. Thus, a subset of inducible LAP-positive Foxp3^−^CD4^+^ Tregs suppressed allergic inflammation in mice [25]–[27]. Gandhi et al. reported that this new Treg population, isolated from human peripheral blood, suppressed the proliferation of other T cells in vitro, which was partly mediated by TGF-β and IL-10 [28]. Our previous study has revealed that the population of CD4^+^LAP^+^ cells was increased in the peripheral blood of colorectal cancer (CRC) patients; moreover, these cells demonstrated a TGF-β–dependent suppressive phenotype [29]. Importantly, we observed a modest increase in CD4^+^Foxp3^+^LAP^+^ T cells in CRC patients, whereas these Tregs were rarely observed in healthy individuals. Chen et al. obtained similar results in a murine experimental autoimmune encephalitis (EAE) model, where a unique subset of LAP-expressing CD4^+^CD25^+^ Tregs exhibited more potent suppressive ability than LAP-negative Tregs [30]. The existence of CD4^+^CD25^+^LAP^+^ Tregs with suppressive activity was also observed in autoimmune syndrome of scurfy mice [31]. However, in humans, the immunomodulatory role of LAP-expressing CD4^+^Foxp3^+^ Tregs is not well characterized. In this work, we demonstrated that CD4^+^Foxp3^+^LAP^+^ Treg population was modestly increased in the peripheral blood of CRC patients and presented a distinct subset of activated Tregs that potently suppressed effector cells through a TGF-β–related mechanism. A higher percentage of LAP^+^ Tregs correlated with tumor progression, suggesting that LAP^+^ Tregs play an important role in immune tolerance to CRC.

## Materials and Methods

### Patients

In total, 42 CRC patients and 21 healthy donors were enrolled in this study. The 42 CRC patients were newly diagnosed with the disease and underwent colectomy without pre-operative chemotherapy and/or radiotherapy at Chang Gung Memorial Hospital, Linkou branch, Taoyuan, Taiwan. Blood samples were collected from the patients by venipuncture before surgery. Patients' clinical characteristics including tumor pathology, CRC stage, and carcinoembryonic antigen levels were retrieved from the medical records.

### Blood and tissue samples

Peripheral blood mononuclear cells (PBMCs) were isolated by Ficoll-Paque Plus density gradient centrifugation method [12]. Lymphocytes were also isolated from the resected specimens of both tumor and non-tumor tissues. Non-tumor tissues were selected from the resection margin of the specimen and confirmed to be non-cancerous by pathological examination. The tumor and non-tumor tissues were finely sliced and crushed, incubated with 0.1% collagenase D (Sigma-Aldrich, USA) in HBSS for 30 min at 37°C and filtered through a nylon mesh. Single cell suspension was prepared with Ficoll (Pharmacia, USA), and leukocytes were separated from the interphase, as previously described [29].

### Ethics statement

Signed informed consent was obtained from all participants before enrollment. The study protocol was designed in accordance with the ethical guidelines of the 2008 Declaration of Helsinki and was approved by the Institutional Review Board of Chang Gung Memorial Hospital, Taiwan.

### Antibodies and reagents

Freshly obtained human lymphocytes were stained with the following fluorescent antibodies against human leukocyte surface markers: CD4-peridinin-chlorophyll-cyanine 5 (PerCP-Cy5.5), CD25-phycoerythrin (PE) or -allophycocyanin (APC), CD45RA-fluorescein isothiocyanate (FITC), CCR7-PE, CCR5-FITC, CCR4-PE-Cy7, CD62L-PE, Ki67-PE or -APC from (BD Biosciences, USA), and LAP (PE and APC) from (R&D Systems, USA). Intracellular staining with Foxp3-APC or -FITC and CTLA-4-APC antibodies was performed after treatment with fixation and permeabilization buffers (eBiosciences, USA), according to the manufacturer's protocol. Intracellular staining for granzyme B, perforin, and TGF-β was performed after stimulation with PMA and ionomycin for 5 h with Golgi stop. The stimulated cells were first reacted with antibodies against the surface markers CD4 and LAP, then fixed and permeabilized with Cytofix/Cytoperm buffer (BD Biosciences, USA), and finally stained with TGF-ß-PE, granzyme B-FITC, or perforin-PE antibodies. The fluorescence intensity was analyzed using BD FACSCalibur (BD Biosciences, USA) and FlowJo software (Tree Star, USA).

### CD4^+^CD25^+^LAP^+^ T cells suppression assay

CD4^+^CD25^+^LAP^+^, CD4^+^CD25^+^LAP^−^, and CD4^+^CD25^−^ T cells from CRC patients were isolated with more than 90% purity by cell sorting, using FACS Aria (BD Biosciences, USA). CD4^+^CD25^+^LAP^+^ and CD4^+^CD25^+^LAP^−^ T cells were mixed with responder T cells (CD4^+^CD25^−^) at a ratio of 1∶1 and activated with anti-CD3 and anti-CD28 antibodies for 96 h. Cells were pulsed with thymidine [^3^H] for 16 h after the stimulation.

### Statistical analysis

The differences between the groups were assessed by Mann–Whitney U test, *t*-test, paired *t*-test, or one-way ANOVA. P values less than 0.05 (*P<0.05; **P<0.01; ***P<0.001) were considered statistically significant. The statistical analyses were performed using GraphPad Prism 5.0 software (GraphPad Software, USA).

## Results

### A group of CD4^+^Foxp3^+^ Tregs preferentially expresses LAP in CRC patients

**Figure 1:**
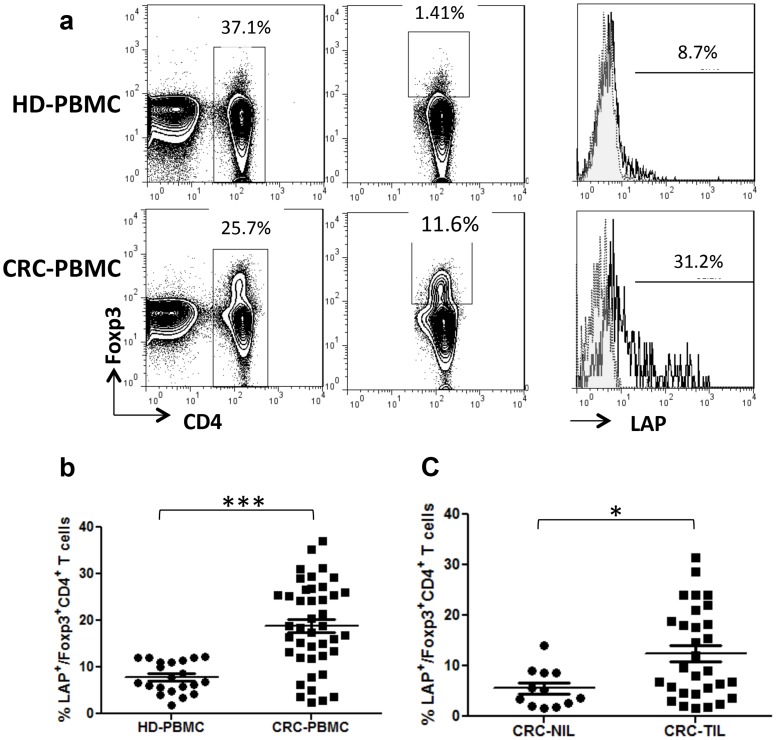
Increased frequency of CD4^+^Foxp3^+^LAP^+^ T cells in peripheral blood and tumor tissues of colorectal cancer patients. (A) The percentage of LAP^+^ cells in the gated CD4^+^Foxp3^+^ subpopulations of colorectal cancer (CRC) patients and healthy donors. Compared to healthy donors, CRC patients showed an increased frequency of LAP^+^CD4^+^Foxp3^+^ T cells in peripheral blood mononuclear cells (PBMCs) and tumor-infiltrating lymphocytes (TILs). (B). PBMCs from CRC patients and healthy donors were isolated, and the percentage of LAP^+^CD4^+^Foxp3^+^ Tregs was calculated by FACS analysis. The difference between samples was analyzed by Student's *t*-test (***P<0.001). (C). Lymphocytes were isolated from tumor and non-tumor tissues of CRC patients, and the percentage of LAP^+^CD4^+^Foxp3^+^T cells was calculated by FACS analysis. CRC-TILs, tumor-infiltrating lymphocytes; CRC-NILs, non-tumor tissue-infiltrating lymphocytes. The difference in the proportion of CD4^+^Foxp3^+^LAP^+^ T cells between CRC-NIL and CRC-TIL populations was analyzed by Student's *t*-test (*P<0.05, **P<0.01, and ***P<0.001).

We first investigated the expression of surface LAP on CD4^+^Foxp3^+^ Tregs isolated from CRC patients. The reliability of LAP staining was confirmed by isotype control monoclonal and recombinant LAP (rLAP) monoclonal antibodies. (Figure S1) The comparison between CRC patients and healthy donors (HD) revealed that LAP expression in CD4^+^Foxp3^+^ T cells isolated from PBMCs was significantly increased in cancer patients (CRC: 18.8%±9.2% vs. HD: 7.8%±3.3%; Fig. 1A and B). Furthermore, Fig 1C shows that the percentage of CD4^+^Foxp3^+^LAP^+^ T cells was significantly higher in tumor tissues than in non-tumor tissues of CRC patients (12.4%±8.8% vs. 5.5%±3.8%, P  =  0.02). The results suggest that the increased population of CD4^+^Foxp3^+^LAP^+^ Tregs in CRC may play an important role in regulating anti-tumor immune response.

**Figure 2:**
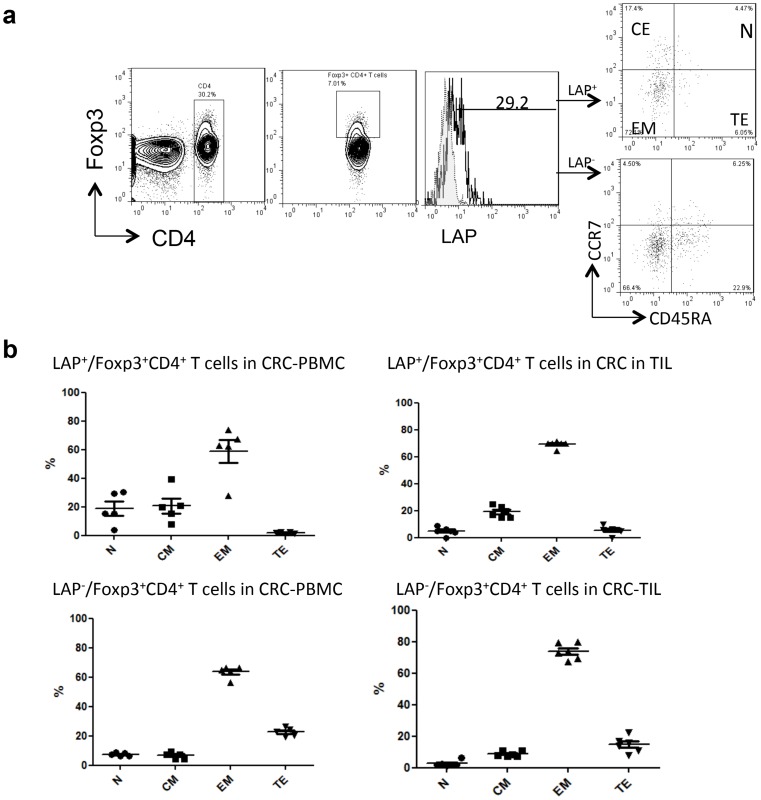
Phenotypic differentiation stages of CD4^+^Foxp3^+^LAP^+^ T cells. PBMC and TIL suspensions were analyzed for the expression of phenotypic markers CCR7 and CD45RA to evaluate the differentiation stage of CD4^+^Foxp3^+^LAP^+^ T cells. (A) Dot blot represents the naïve (N, CD45RA^+^CCR7^+^), central memory (CM, CD45RA^−^CCR7^+^), effector memory (EM, CD45RA^−^CCR7^−^), and terminal effector (TE, CD45RA^+^CCR7^−^) CD4^+^ Foxp3^+^LAP^+^ Tregs. (B) Differentiation stages of CD4^+^Foxp3^+^LAP^+^ Tregs in the peripheral blood, tumor tissues, and non-tumor tissues of CRC patients were determined by flow cytometry. Each dot represents the mean ± SD of an individual sample.

The phenotype of CD4^+^Foxp3^+^ Tregs of CRC patients was analyzed based on the expression of surface memory cell markers CD45RA and CCR7 (Fig. 2A). CD45RA and CCR7 expression patterns indicated that both LAP^+^ and LAP^−^ subsets of Foxp3^+^CD4^+^ T cells mostly had an effector/memory phenotype in peripheral blood as well as in tumor sites of CRC patients (Fig. 2B) [17]. Thus, LAP-positive Tregs in CRC did not differ from their LAP-negative counterparts in the expression of the effector/memory phenotype.

### Elevated expression of activated Treg-related markers in CD4^+^Foxp3^+^LAP^+^ T cells

**Figure 3:**
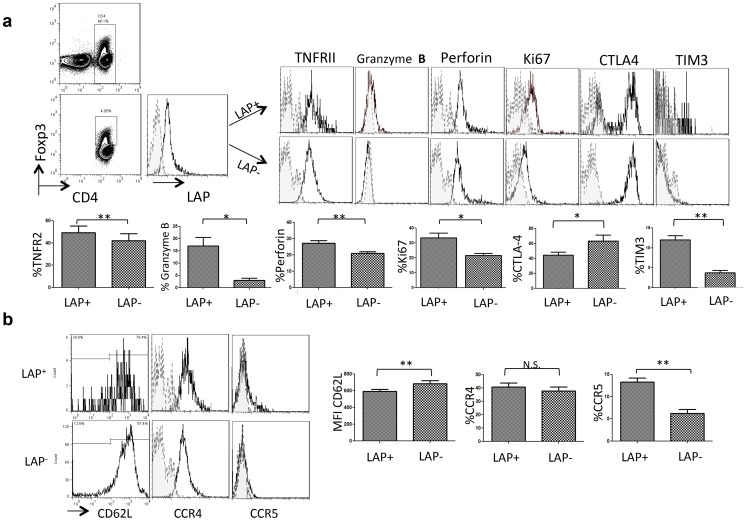
Elevated expression of activated Treg-related molecules in CD4^+^Foxp3^+^LAP^+^ T cells. (**A**) PBMCs of CRC patients were analyzed for the expression of Treg markers TNFR II, granzyme B, perforin, Ki67, CTLA-4, and Tim-3 by FACS. The histogram represents the expression of Treg markers in CD4^+^Foxp3^+^LAP^+^ Tregs and CD4^+^Foxp3^+^LAP^−^ Tregs; the expression difference between T cell populations was analyzed by paired *t*-test (*P<0.05, **P<0.01, and ***P<0.001). (B) Typical histograms represent the percentage of CD62L-, CCR4-, and CCR5-positive CD4^+^Foxp3^+^LAP^+^ Tregs and CD4^+^Foxp3^+^LAP^−^ Tregs; the expression levels of CD62L, CCR4, and CCR5 were compared between both T cell populations. The data were analyzed by paired *t*-test (*P<0.05, **P<0.01, and ***P<0.001).

We examined the expression of Treg-associated activating/effector molecules, including TNFR super family members, granzyme B, perforin, Ki67, CTLA-4, and Tim-3, in CD4^+^Foxp3^+^ T cells. Compared to CD4^+^Foxp3^+^LAP^−^ T cells, CD4^+^Foxp3^+^LAP^+^ T cells showed significantly higher expression of TNFRII, granzyme B, perforin, Ki67, and Tim-3 and lower expression of CTLA-4 (Fig. 3A). These data indicate that in CRC, an increased proportion of LAP-positive CD4^+^Foxp3^+^ Tregs has an activated proliferating phenotype.

Several lines of evidence from mouse and human studies suggest that the expression of certain chemokine receptors, including CCR4 and CCR5, is characteristic for an activated subgroup of CD4^+^Foxp3^+^ Tregs. The expression analysis of CCR4, CCR5, and L-selectin (CD62L) indicated that CD62L was significantly downregulated in CD4^+^Foxp3^+^LAP^+^ T cells compared to their LAP^−^ negative counterparts (mean fluorescence intensity (MFI), 594±41.9 vs. 684±70.9, P = 0.006); the expression of CCR4 was similar, whereas that of CCR5 increased in CD4^+^ Foxp3^+^LAP^+^ T cells compared to CD4^+^Foxp3^+^LAP^−^ T cells (CCR4: 40.7%±5.9% vs. 37.7%±6.2%, P = 0.2; CCR5: 13.2%±1.9% vs. 6.2%±1.7%, P = 0.001). These results indicate that CD4^+^ Foxp3^+^LAP^+^ Tregs have an activated phenotype.

### CD4^+^Foxp3^+^LAP^+^ Tregs exhibit potent TGF-β–dependent suppression ability

**Figure 4:**
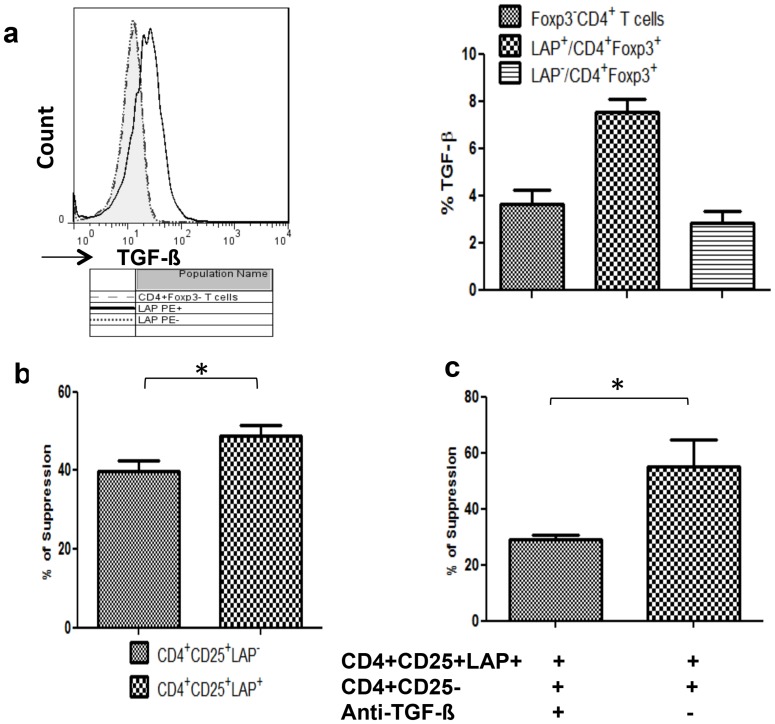
CD4^+^Foxp3^+^LAP^+^ Tregs exhibit potent suppression ability in a TGF-β–dependent manner. (A) The expression of TGF-β in CD4^+^Foxp3^+^LAP^+^, CD4^+^Foxp3^+^LAP^−^, and CD4^+^Foxp3^+^ Tregs was analyzed by FACS. T cells were stimulated in vitro as described in the Materials and methods, and TGF-β expression was compared between these three groups by paired *t*-test (**P<0.01). (B) CD4^+^CD25^+^LAP^+^ T cells and CD4^+^CD25^+^LAP^−^ T cells were separated by cell sorting using FACSAria and stimulated with anti-CD3 and anti-CD28 antibodies for 96 h. T cell proliferation was assessed by thymidine [^3^H] incorporation and expressed as the mean ± SD of three independent experiments; the data were analyzed by paired *t*-test (*P<0.05). (C) Anti-TGF-β antibody (10 µg/mL) was added to CD4^+^CD25^+^LAP^+^ Tregs co-cultured with CD4^+^CD25^−^LAP^+^ T cells (ratio of 1∶1) and activated by anti-CD3 and anti-CD28 mAbs. The percentage of suppression in the presence or absence of anti-TGF-β mAb is expressed as the mean ± SD of three independent experiments. *P<0.05 by paired *t*-test.

TGF-β is an immunosuppressive cytokine known to play a critical role in the generation and functional activity of Tregs. Membrane TGF-β is maintained in a latent form by non-covalent binding to LAP. To examine the association of the LAP-positive Treg phenotype with TGF-β, we analyzed TGF-β production in CRC Foxp3^+^LAP^+^ Tregs stimulated with PMA/ionomycin. As shown in Fig. 4A, TGF-β levels were higher in the LAP-positive Treg subset than in the LAP-negative Treg subset (7.50%±1.1% vs. 2.8%±1.1%, P = 0.001), suggesting that CD4^+^Foxp3^+^LAP^+^ cells exert their regulatory function through the TGF-β pathway.

Next, we evaluated the expression of LAP among CD4^+^CD25^−^, CD4^+^CD25^lo^, and CD4^+^CD25^hi^ subpopulations of CD4^+^ Tregs and found that in CRC patients, LAP-positive CD4^+^Foxp3^+^ Tregs were predominantly observed within the CD4^+^CD25^hi^ compartment. Then, CD4^+^CD25^+^, CD4^+^CD25^−^, CD4^+^CD25^+^LAP^+^, and CD4^+^CD25^+^LAP^−^ Treg populations were separated by cell sorting, and their ability to inhibit the proliferation of freshly isolated CD4^+^CD25^−^ T cells was evaluated by the [H^3^] thymidine incorporation assay. Among these Treg subpopulations, CD4^+^CD25^+^LAP^+^ cells displayed the most potent suppressive ability (P<0.03; Fig. 4B), which was inhibited in vitro by the administration of anti-TGF-β antibody (Fig. 4C). Enrichment in peripheral blood CD4^+^Foxp3^+^LAP^+^ T cells correlated with CRC progression.

**Figure 5:**
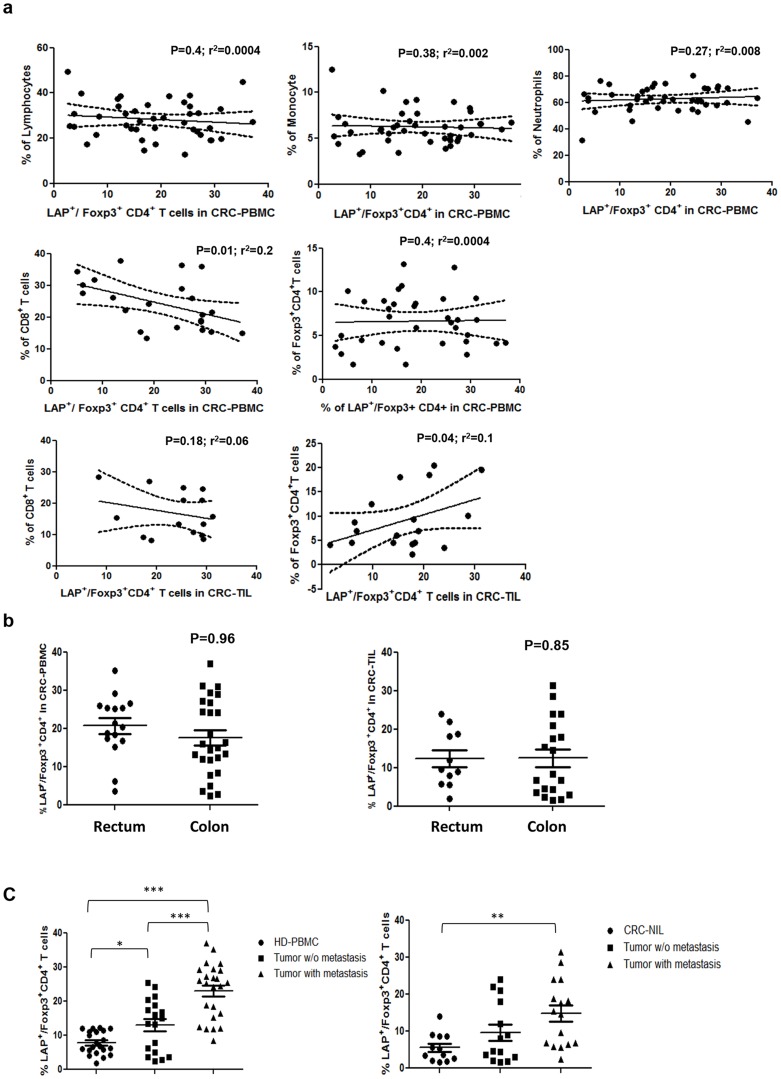
Enrichment of CD4^+^Foxp3^+^LAP^+^ T cells in peripheral blood correlates with colorectal cancer progression. (A) Correlation between LAP^+^Foxp3^+^CD4^+^ T cells and CD4^+^Foxp3^+^ T cells, CD8^+^ T cells, total lymphocytes, monocytes, and neutrophils (r^2^ and P values) was evaluated by the Pearson correlation method. (B) The percentage of LAP^+^ Tregs in PBMC and TIL populations was compared between CRC patients with rectum and colon tumors; the data were analyzed by paired *t*-test (*P<0.05). (C) CRC patients were grouped based on the presence or absence of lymph node or distant metastases. The percentage of CD4^+^Foxp3^+^LAP^+^ Tregs in PBMC and TIL populations was compared among the patient groups with different CRC stages and the healthy donors. Statistical analysis was performed by one-way ANOVA (**P<0.05, **P<0.01, and ***P<0.001).

The clinical relevance of CD4^+^Foxp3^+^LAP^+^ T cells in CRC was examined in 42 CRC patients (their demographic and clinical characteristics are presented in Table 1). Only 9 patients had recurrent cancer or disease progression. The percentage of CD4^+^Foxp3^+^LAP^+^ T cells in the CD4^+^Foxp3^+^ T cell population did not correlate with that of CD4^+^Foxp3^+^ T cells in the CD4^+^ T cell population in peripheral blood. In tumor-infiltrating lymphocyte (TIL) population, the percentage of CD4^+^Foxp3^+^LAP^+^ T cells correlated with that of CD4^+^Foxp3^+^ T cells (r^2^ = 0.1, P = 0.04). The proportion of CD4^+^Foxp3^+^LAP^+^ T cells inversely correlated with that of CD8^+^ T cells in peripheral blood (r^2^ = 0.2, P = 0.01); in TIL, a trend of inverse correlation between CD4^+^Foxp3^+^LAP^+^ and CD8^+^ T cells was observed; however, it was not statistically significant (r^2^ = 0.08, P = 0.18). Besides, we found no correlation between circulating CD4^+^Foxp3^+^LAP^+^ T cells and other inflammatory cells such as neutrophils, lymphocytes, and monocytes (Fig. 5A). The study included 26 patients with colon cancer and 16 patients with rectal cancer; however, the percentage of circulating or tumor infiltrating CD4^+^Foxp3^+^LAP^+^ T cells in the CD4^+^Foxp3^+^ T cell population did not differ between the two groups of CRC patients (Fig 5B).

**Table 1:** Patients' demographic and clinical characteristics.

	Number	%
Age (range and median)	54–85 (67)	
Sex		
Female	23	54.8
Male	19	45.2
Tumor		
Ascending	4	9.5
Transverse	8	19
Descending	1	2.5
Sigmoid	13	31
Rectum	16	38
Stage (AJCC TNM)		
I	1	2.4
II	7	16.7
III	26	62
IV	8	19
Carcinoembryonic antigen (ng/dL) median (range)	12.9 (<0.5–215.4)	

Finally, we investigated the association of CD4^+^Foxp3^+^LAP^+^ T cells with tumor stage. As shown in Fig. 5C, the percentage of CD4^+^Foxp3^+^LAP^+^ T cells in the peripheral blood of metastatic patients was significantly higher than that in CRC patients without metastasis and in healthy donors. Furthermore, the size of CD4^+^Foxp3^+^LAP^+^ Treg population was increased in tumor tissues compared to non-tumor tissues (Fig 5C). Thus, the increase of LAP^+^ Tregs in the peripheral blood of CRC patients could reflect the increase of LAP^+^ Tregs in tumor tissues and could be correlated with the cancer stage, suggesting that the size of peripheral blood LAP-positive CD4^+^Foxp3^+^ T cell subpopulation can serve as a surrogate marker for the immunosuppression process in CRC.

## Discussion

The results of our study indicate that LAP expression marks a subpopulation of CD4^+^Foxp3^+^ Tregs with potent suppressive ability. Although most of CD4^+^Foxp3^+^ Tregs in CRC patients are classified as activated, among them, LAP-positive Tregs have even more potent suppressive functions. This is manifested by higher expression of activated cell markers, increased production of immunosuppressive TGF-β, and more potent suppressive ability in vitro in LAP-positive Tregs compared to LAP-negative Tregs. Our findings suggest that LAP can be a potential marker to identify a subgroup of Tregs that exhibit TGF-β–related suppressive functions. This Treg subpopulation was enriched in the peripheral blood of CRC patients, which correlated with tumor progression, suggesting that CD4^+^Foxp3^+^LAP^+^ Tregs may have clinical application as markers of tumor-specific Tregs in CRC patients.

The functional role of LAP in Tregs is not clear. LAP remains noncovalently associated with TGF-β after cleavage from the precursor peptide and form the inactive latent TGF-β complex; therefore, it contributes to the prevention of uncontrolled activation of TGF-β receptors. Nakaruma et al. were the first to demonstrate that both murine and human CD4^+^CD25^+^ Tregs could exert TGF-β–dependent suppression of effector cells, which was reversed by the recombinant LAP. They also demonstrated that LAP-positive CD4^+^ T cells could suppress colitis in vivo, suggesting, for the first time, that LAP could be considered as a regulatory marker [24]. Qida et al. further demonstrated that LAP expression on murine CD4^+^ T cells was induced by TGF-β independently of Foxp3 [25]. Shevach et al. observed the expression of TGF-β-LAP complexes on the activated Tregs but not on the resting Tregs induced via non-antigen stimulation [21], [31], [32]. LAP^+^ Tregs suppressed the proliferation of activated T cells but not of naïve T cells in a TGF-β–dependent manner and mediated infection tolerance by converting Foxp3-negative Tregs to functionally active Foxp3-positive Tregs [32]. It is believed that the LAP-TGF-β complex represents an important mechanism for Tregs to maintain immune homeostasis via TGF-β signaling.

Chen et al. have further functionally characterized CD4^+^CD25^+^LAP^+^ Tregs in a murine autoimmune encephalitis (EAE) model [30]. CD4^+^CD25^+^LAP^+^ Treg population had higher frequency of Foxp3 expression and produced increased levels of effector molecules and cytokines than their LAP-negative counterparts. LAP-expressing Tregs were anergic and suppressive in vitro; however, their suppressive activity was more potent compared to that of other Treg types. These cells expressed both TGF-β and its receptors; the suppression ability of LAP^+^ Tregs was reversed by TGF-β neutralization, indicating that CD4^+^CD25^+^LAP^+^ Tregs were, at least in part, regulated in a TGF-β–dependent manner. Furthermore, in vivo adoptive transfer of CD4^+^CD25^+^LAP^+^ Tregs ameliorated EAE, confirming the immunosuppressive potency of this Treg population. Based on these findings, LAP could be considered as a marker for the identification of a subgroup of CD4^+^CD25^+^ Tregs with potent suppressive ability [30]. In humans, Shevach et al. showed that the presence of surface TGF-β complexed with LAP induced Foxp3 and resulted in the suppression of responder cells, indicating a possibility that both Foxp3 and LAP could confer more potent suppressive activity on human Treg cell population [31].

Even though the role of LAP-positive Tregs has not been fully elucidated, the available data suggest that LAP can be a useful biomarker to identify activated Tregs in mice and humans. In mice, LAP can be applied for the in vitro selection of expanded CD4^+^Foxp3^+^ suppressive Tregs [33], while in humans, it has been suggested as a marker for monitoring Tregs after anti-CTLA-4 immunotherapy [34]. Our study further strengthens the notion that LAP expression can distinguish activated Tregs, especially the ones circulating in the peripheral blood of CRC patients. Most importantly, tumor progression correlated with the increased proportion of LAP^+^ Tregs. Taken together, these data suggest that circulating LAP-positive CD4^+^ Foxp3^+^ Tregs, rather than the general population of CD4^+^Foxp3^+^ T cells, can be used as a more accurate and specific marker for monitoring the immunological status of cancer patients.

In conclusion, CD4^+^ Tregs that express both Foxp3 and LAP exhibit higher immunosuppressive activity than other Treg cell subsets. The suppressive LAP^+^CD4^+^Foxp3^+^ Tregs regulate immune responses, at least in part, via TGF-β signaling. The enrichment of CD4^+^Foxp3^+^LAP^+^ Tregs in the peripheral blood of CRC patients can be potentially used for monitoring cancer immunotherapy in clinical settings.

## Supporting Information

Figure S1 **Expression of LAP.** (A). The isotype control monoclonal antibody (IC) and recombinant LAP (rLAP) was confirmed the reliability of staining. (B). Identification of CD4^+^Foxp3^+^ T cells. The isotype control monoclonal antibody (IC) was used to confirm the LAP^+^ T cells staining.
(TIF)
