# Supplementary figures and images for: CD4+ T Cells Expressing Latency-Associated Peptide and Foxp3 Are an Activated Subgroup of Regulatory T Cells Enriched in Patients with Colorectal Cancer
Source: PLoS One. 2014 Sep 30;9(9):e108554. doi: 10.1371/journal.pone.0108554 (PMC4182495; doi:10.1371/journal.pone.0108554)

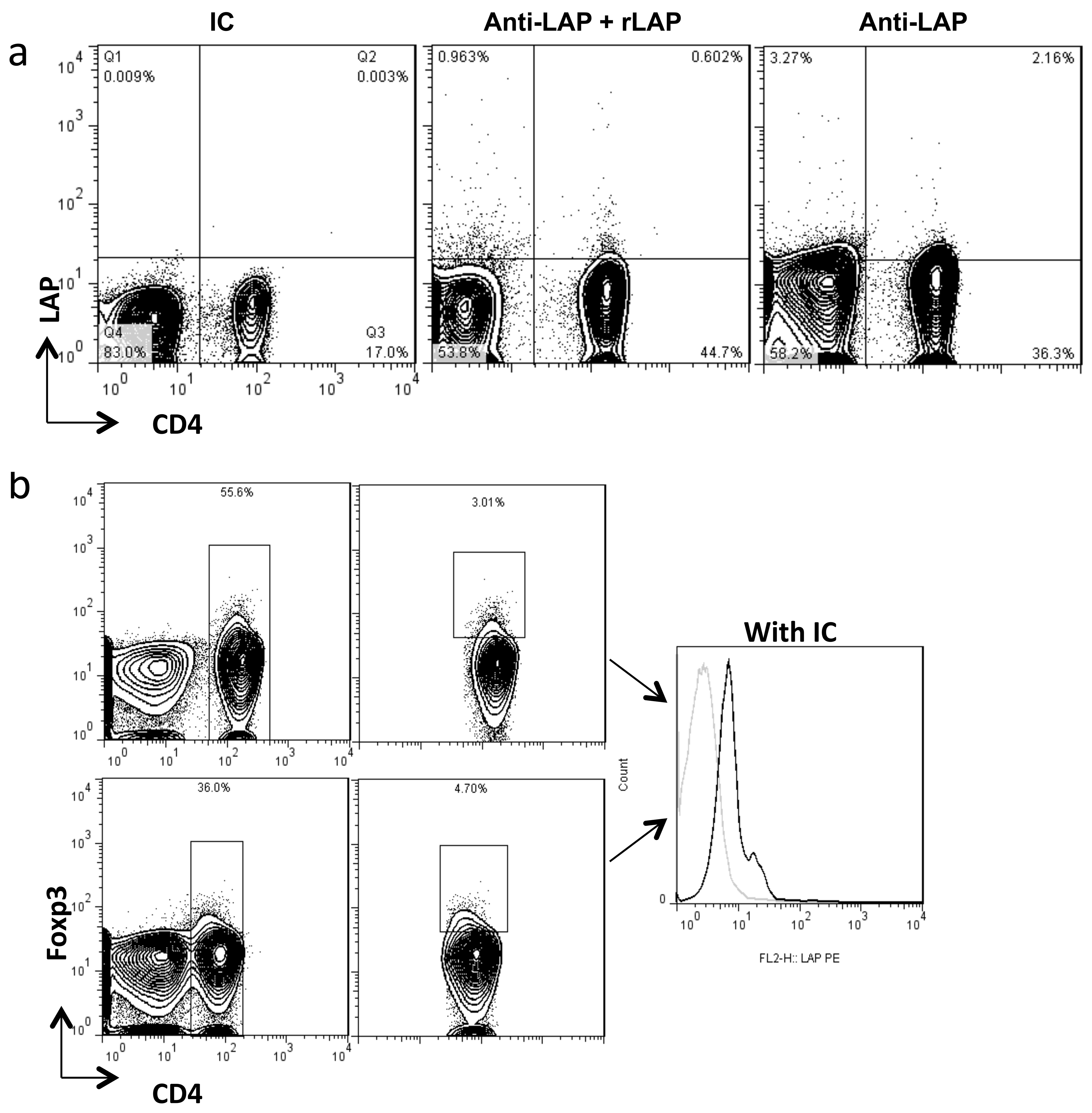

Supplement: Figure S1 — Expression of LAP. (A). The isotype control monoclonal antibody (IC) and recombinant LAP (rLAP) was confirmed the reliability of staining. (B). Identification of CD4+Foxp3+ T cells. The isotype control monoclonal antibody (IC) was used to confirm the LAP+ T cells staining. (TIF) [file pone.0108554.s001.tif]
